# Newly identified miRNAs may contribute to aerenchyma formation in sugarcane roots

**DOI:** 10.1002/pld3.204

**Published:** 2020-03-26

**Authors:** Eveline Queiroz de Pinho Tavares, Marina Camara Mattos Martins, Adriana Grandis, Grayce H. Romim, Amanda Rusiska Piovezani, Jonas Weissmann Gaiarsa, Marcos Silveira Buckeridge

**Affiliations:** ^1^ Departamento de Botânica Instituto de Biociências Universidade de São Paulo São Paulo Brazil; ^2^ Centro de Facilidades Para a Pesquisa Instituto de Ciências Biomédicas Universidade de São Paulo São Paulo Brazil

**Keywords:** aerenchyma, bioenergy, cell wall, miRNA, sugarcane

## Abstract

Small RNAs comprise three families of noncoding regulatory RNAs that control gene expression by blocking mRNA translation or leading to mRNA cleavage. Such post‐transcriptional negative regulation is relevant for both plant development and environmental adaptations. An important biotechnological application of miRNA identification is the discovery of regulators and effectors of cell wall degradation, which can improve/facilitate hydrolysis of cell wall polymers for second‐generation bioethanol production. The recent characterization of plant innate cell wall modifications occurring during root aerenchyma development triggered by ethylene led to the possibility of prospection for mechanisms of cell wall disassembly in sugarcane. By using next‐generation sequencing, 39 miRNAs were identified in root segments along the process of aerenchyma development. Among them, 31 miRNAs were unknown to the sugarcane miRBase repository but previously identified as produced by its relative *Sorghum bicolor.* Key putative targets related to signal transduction, carbohydrate metabolic process, and cell wall organization or biogenesis were among the most representative gene categories targeted by miRNA. They belong to the subclasses of genes associated with the four modules of cell wall modification in sugarcane roots: cell expansion, cell separation, hemicellulose, and cellulose hydrolysis. Thirteen miRNAs possibly related to ethylene perception and signaling were also identified. Our findings suggest that miRNAs may be involved in the regulation of cell wall degradation during aerenchyma formation. This work also points out to potential molecular tools for sugarcane improvement in the context of second‐generation biofuels.

## 1 INTRODUCTION

miRNAs are a class of relevant plant development regulators (Borges & Martienssen, 2015; Chen, 2004; Guleria & Yadav, 2011). Post‐transcriptional regulation is achieved through sequence‐specific base‐pairing of 20 to 22 nucleotides of a small RNA and a mRNA. Dicer‐like proteins are plant ribonucleases responsible for processing primary miRNAs leading to its mature functional conformation. Within the cytoplasm, the active guide strand (i.e., the one that effectively binds to the target mRNA) is then separated from its complementary strand (named miRNA* or star miRNA). miRNA* is degraded and mature miRNA is loaded onto the argonaute protein, giving rise to the RNA‐induced silencing complex (RISC). This protein complex leads the miRNA guide strand to target mRNAs due to base complementarity within its 3’ UTR sequence (Li & Zhang, 2016). The mRNA can be then targeted for degradation, or its translational process can be blocked (Borges & Martienssen, 2015).

The wide range of pathways regulated by miRNA within cellular metabolism has led to the assumption that plant miRNA can serve as important tools for plant improvement (Zhang, 2015). miRNA identification involved in plant tolerance to environmental stresses and carbohydrate metabolism can be especially relevant for crops, since increased biomass, higher digestibility, and stress tolerance are desirable traits, especially for biofuel industries. Some of these crops are *Panicum virgatum*, *Sorghum bicolor, Zea mays,* and *Saccharum* sp. For instance, *P. virgatum* plants overexpressing *Z. mays* miR156 displayed distinct biomass composition and presented higher saccharification yields due to increased starch content (Chuck et al., 2011; Fu et al., 2012). Data mining for miRNA regulation among cell wall‐related transcripts in *Sorghum bicolor* has led to the identification of 10 putative targets, including laccases and cellulose synthases (Rai et al., 2016). However, the underlying miRNA regulation of cell wall metabolism in sugarcane lacks experimental evidence.

Four miRNA experimental procedures have already been performed with sugarcane miRNA using leaves (Ferreira et al., 2012; Thiebaut et al., 2014; Thiebaut, Rojas, et al., 2012; Zanca et al., 2010), buds (Ortiz‐Morea et al., 2013; Zanca et al., 2010), roots (Thiebaut et al., 2014; Thiebaut, Rojas, et al., 2012), and whole plant (Thiebaut, Rojas, et al., 2012), and a sugarcane EST database (Vettore, Silva, Kemper, & Arruda, 2001) was also subjected to miRNA identification (Ortiz‐Morea et al., 2013). Surprisingly, only 19 miRNA precursors and 20 mature sugarcane sequences are currently available in the miRBase repository v.21, which represents less than 1% of monocot miRNAs (release April 21, 2017; http://www.mirbase.org/index.shtml). It inevitably underestimates sugarcane miRNA diversity, and it also represents the need for further investigation of miRNA function and identification. Sugarcane features directly related to bioenergy, such as cell wall composition and modifications, lack miRNA regulation studies.

Sugarcane is an essential crop in Brazil due to its widespread use in sugar and ethanol production. Also, further expansion of production is possible without need to use land presently occupied by preserved biomes or food production as projected to 2045 (Jaiswal et al., 2017). Sugar is obtained through culm milling followed by sucrose extraction, whereas ethanol results from sugar fermentation. Fermentation can occur using two different substrates, which defines first‐generation (1G) and second‐generation (2G) ethanol. The fermentation of the sucrose stored within sugarcane culm has supported Brazil as the second larger 1G producer in the world. Although available commercially, 2G ethanol production still needs further improvements, since it requires more effective depolymerization of structural polysaccharides of the cell wall (Buckeridge & De Souza, 2017).

Recently, the characterization of aerenchyma formation within sugarcane roots uncovered the potential use of innate pathways for cell wall deconstruction, facilitating the access to cell wall polymers and paving the way toward successful 2G ethanol production (Leite et al., 2017; Tavares, Souza, & Buckeridge, 2015). Lysigenous aerenchyma is a developmental process activated by the hormone ethylene. Its action leads to the opening of gas spaces within parenchymatic tissues due to programmed cell death and cell wall modifications (Takahashi, Yamauchi, Colmer, & Nakazono, 2014; Tavares et al., 2018; Yamauchi, Colmer, Pedersen, & Nakazono, 2018; Yamauchi, Shimamura, Nakazono, & Mochizukic, 2013). Immunolocalization showed arabinoxylan debranching, homogalacturonan hydrolysis from the middle lamella, and β‐glucan mobilization during the formation of aerenchyma in sugarcane roots (Leite et al., 2017). The negative regulation played upon homogalacturonan hydrolysis by an ethylene response factor, RAV1, represents an important trigger to cell wall attack (Rahji et al., 2011; Tavares et al., 2019). Thus, aerenchyma formation seems to rely on cell targeting induced by ethylene and auxin balance. This is followed by cell expansion and separation, programmed cell death, and hemicellulose and cellulose hydrolysis. Each step configures a conserved set of pathways—named “modules”—shared between other endogenous cell wall degradation events (Grandis, Souza, Tavares, & Buckeridge, 2014; Tavares et al., 2015). Although cell wall changes are subtle and sugarcane pectin content is rather low (De Souza, Leite, Pattathil, Hahn, & Buckeridge, 2013), the effects of partial pectin degradation within plant tissues, especially in the middle lamella, may be quite relevant for saccharification and bioenergy production (Latarullo, Tavares, Maldonado, Leite, & Buckeridge, 2016). During aerenchyma formation in sugarcane roots, pectin degradation is thought to be a result of the attack by acetyl esterases, endopolygalacturonases, β‐galactosidases, and α‐arabinofuranosidases, followed by the action of β‐glucan‐/callose‐hydrolyzing enzymes (Grandis et al., 2019; Tavares et al., 2018, 2019). Concomitantly, there are modifications in arabinoxylan (by α‐arabinofuranosidases), xyloglucan (by xyloglucan endotransglucosylase/hydrolase), xyloglucan–cellulose interactions (by expansins), and partial hydrolysis of cellulose (Grandis et al., 2019). Thus, the precise control underlying this process might be a key factor to understand modulation of cell wall changes in sugarcane. One question is to what extent epigenetics might be involved in the control of aerenchyma formation in sugarcane and other grasses.

In this study, we identified 39 expressed miRNAs within sugarcane roots undergoing aerenchyma formation. Transcripts related to carbohydrate metabolic process and cell wall organization or biogenesis were predicted to be targeted by the sequenced miRNA. Among those, miRNAs expressed during aerenchyma formation and presumably target transcripts related to several cell wall polysaccharides previously detected in sugarcane (Leite et al., 2017). A more significant fraction of these transcripts is related to pectin degradation. The diversity of miRNA targeting pectin‐related and ethylene regulation‐related transcripts corroborates previous data showing decreasing cellular adhesion and hormone signaling as fundamental mechanisms during the onset of aerenchyma formation (Grandis et al., 2019; Leite et al., 2017; Tavares et al., 2018, 2019). Our results represent an important step toward the identification of miRNAs involved in sugarcane cell wall degradation, as well as its hormonal control. It also paves the way for the development of biotechnology in the biofuel field.

## 2 MATERIALS AND METHODS

### 2.1 Plant samples and RNA extraction

**Figure 1:**
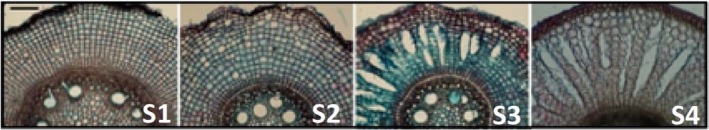
Aerenchyma formation in sugarcane roots from apex to 4 cm divided into 1 cm segments (S1–S4). Sections were stained with 1% safrablau (Bukatsch, 1972). Scale bar = 20 µm

All experiments employed sugarcane (*Saccharum* sp.) variety SP80‐3280 grown in Piracicaba, São Paulo, Brazil. Lateral buds from harvested culms were planted in vermiculite and grown for 4 months supplied with 40 g NPK (30:20:30) fertilizer. Plants were watered weekly with 100 ml of water and grown under natural environmental conditions including periodic rain and natural South Hemisphere summer climate fluctuations (from December 2013 until March 2014). Thirty plants were pooled in three biological replicates, with ten plants each. The first 4 cm of tiller roots was collected and separated into 4 one‐centimeter sections from the apex containing the meristem (S1) toward the base (S4) (Leite et al., 2017) (Figure 1).

Total RNA extraction proceeded with 100 mg of each replicate further homogenized using mirVana™ miRNA Isolation Kit (Thermo Fisher Scientific^®^), according to manufacturer's instructions. The same set of samples was used for qRT‐PCR validation, according to Tavares et al. (2019).

### 2.2 miRNA sequencing

RNAs shorter than 200 nt were first enriched by Magnetic Bead Purification Module (Thermo Fisher Scientific^®^) and next used on Ion Total RNA‐Seq Kit v2 (Thermo Fisher Scientific^®^), according to manufacturer's instructions. Samples were sequenced on Ion Proton™ Sequencer, with 500 flows for 200‐base read sequencing. Pools of three bar‐coded sample libraries were loaded onto each Ion PI sequencing chip v2.

### 2.3 Bioinformatic analyses

Sequence quality was first evaluated by using FastQC (Andrews, 2010) with default settings. Ribosome, transfer, small nuclear, small nucleolar, long no‐coding, and trans‐acting small interfering RNAs (rRNA, tRNA, snRNA, snoRNA, lncRNA, and tasiRNA) were removed by Blastn tool. Sequencing reads were mapped by mirDeep2 (Friedländer, Mackowiak, Li, Chen, & Rajewsky, 2012), using *S. bicolor* v.3.1 as reference genome (Paterson et al., 2009). Reads shorter than 17 nt were not used during mapping step. A 250‐nt window around mapped reads was used for precursor prediction and excision. miRNA mature sequences were identified based on sugarcane and *S. bicolor* mature and precursor sequences deposited on miRBase v.21 onto miRDeep2. miRDeep2 tools were manipulated through Galaxy platform 7.221.3 (Afgan et al., 2016).

Precursor sequence displaying A/U percentage outside 30%–70% range (Zhang, Pan, Cox, Cobb, & Anderson, 2006) and those with minimum fold energy index (MFEI) values below 0.7 (Zhang et al., 2006) or below the minimum free energy of folding randomization (computed by Randfold; Bonnet, Wuyts, Rouze, & Peer, 2004) were discarded. Precursor miRNA not present in all biological replicates from the same root segment was not used in the data analysis. miRNA abundance was expressed in terms of reads per million (RPM).

**Figure 2:**
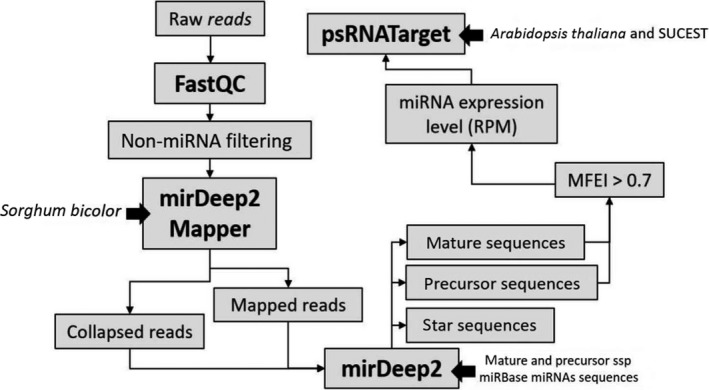
Pipeline for miRNA prediction using mirDeep2 and target identification through psRNATarget. Ion Proton raw reads were uploaded on FastQC for quality analysis, and further, rRNA, tRNA, snRNA, snoRNA, lncRNA, and tasiRNA were removed. Filtered reads were uploaded on miRDeep2 mapper module together with *Sorghum bicolor *as the reference genome. Collapsed and mapped reads output were used as input on miRDeep2 core together with mature and precursor miRNA sequences from *S. bicolor *and *Saccharum *sp retrieved from miRBase v. 21. Precursor, mature, and star miRNA sequences were predicted. The mature miRNA expression level was measured as reads per million if the corresponding precursor presented a minimum fold energy index (MFEI) higher than 0.7. Target prediction was performed by uploading mature miRNA sequences from previous steps and using the sugarcane EST database (SUCEST) and *Arabidopsis thaliana *as queries

Target prediction was performed by using novel and conserved miRNA sequences and the psRNATarget (Dai & Zhao, 2011) tool, with default configurations. Sugarcane ESTs were used as target database (Vettore et al., 2001). Alternatively, *Arabidopsis thaliana* genome (TAIR release 2004/01/22) was also used for target prediction. The experimental design is summarized in Figure 2.

### 2.4 Stem‐loop reverse transcription and qRT‐PCR for target validation

Stem‐loop primers were designed according to Varkonyi‐Gasic, Wu, Wood, Walton, and Hellens (2007). Reverse transcription was performed using SuperScript^®^ III First‐Strand Synthesis System (Thermo Fisher Scientific^®^), with 2 µg of total RNA, 200 ng random hexamers, and 20 µM stem‐loop primer. Primers for target sugarcane ESTs were designed using Primer 3 (http://frodo.wi.mit.edu/primer3/) and the following parameters: Tm 58°C to 60°C, 40%–60% GC, and amplicons ranging from 50 to 150 bp. EST primer efficiencies were calculated on the basis of standard curve dilutions, and only those with efficiency {[10(−1/slope)‐1] × 100} within the 90%–110% range were used.

qRT‐PCR was performed using SYBR Green PCR Master Mix (Thermo Fisher Scientific^®^) according to the manufacturer's instructions. All reactions were made in 3 technical replicates. The cycling amplification consisted of denaturation at 95°C for 5 min, followed by 40 cycles of denaturation at 95°C, primer annealing at 60°C, and extension at 72°C for 1 min each step. Expression ratio was determined by qBase Plus 2.0 software (Biogazelle, Zwijnaarde, Belgium; Hellemans, Mortier, Paepe, Speleman, & Vandesompele, 2007). The reference primers [polyubiquitin (SCCCST2001G02.g), rRNA 60S (SCJFRZ2009G01.g), ubiquitin (SCBGLR1002D06.g, SCCCCL3080A11.g), and actin (SCRFLR1012H05.g)] were selected using the geNormPLUS tool (Biogazelle, Zwijnaarde, Belgium; Vandesompele et al., 2002). An aliquot of treated RNA was used in qRT‐PCR to rule out DNA contamination using GAPDH primer (Iskandar et al., 2004). Sugarcane EST sequences are available at http://sucest-fun.org (Vettore et al., 2001). All primer sequences used in this study are listed in Table S1.

## 3 RESULTS

### 3.1 Root miRNA sequencing and precursor prediction

The development of the lysigenous aerenchyma was observed along the root segments. The root cortex was intact within S1 and S2, and the aerenchyma area was noticeable in S3 by the opening of gas spaces, which increased in S4 (Figure 1). This was in accordance with previous results (Grandis et al., 2019; Leite et al., 2017; Tavares et al., 2018).

Aiming at characterizing sugarcane miRNA transcriptome and identifying candidates related to aerenchyma formation, 4 miRNA libraries were obtained from sequenced RNA extracts from sugarcane roots with developing aerenchyma. A total of 12 libraries (3 biological replicates from 4 root segments) produced 180435212 reads after Ion Proton sequencing, ranging from 73 (S1) to 31.5 (S4) million reads (Table S2). Non‐miRNA filtering (taking out rRNA, tRNA, snRNA, snoRNA, lncRNA, and tasiRNA) led to 119961617 total reads in all libraries.

The remaining sequences after the filtering and trimming steps showed lengths ranging from 20 to 22 nt (Table S3), which is in accordance with the expected range for miRNA (Li & Zhang, 2016). The dominance of 21‐nt length sequences was similar to the tendency observed elsewhere for sugarcane miRNA sequences from four different cultivars (Ferreira et al., 2012) but distinct from the 22‐ and 24‐nt bias shown by other reports with the same plant (Carnavale‐Bottino et al., 2013; Lin, Chen, Qin, & Lin, 2014; Thiebaut, Grativol, et al., 2012; Thiebaut et al., 2014). Furthermore, the majority of mature miRNA presented U as the first nucleotide (Table S3), which is in accordance with previously reported data on sugarcane miRNA (Ferreira et al., 2012; Thiebaut, Rojas, et al., 2012).

### 3.2 Reference genome read mapping and miRNA prediction

The pipeline described above allowed the identification of 39 miRNAs, from 19 different families. Five out of 39 have already been deposited as sugarcane miRNAs in miRBase (Table 1). The remaining 34 miRNAs that have been identified in *S. bicolor* were named on the basis of the observed homology between predicted sugarcane miRNA and those from *S. bicolor* available on miRBase.

**Table 1:** Sugarcane miRNA families, mature sequence, and genomic locations according to *Sorghum bicolor *reference genome

miRNA family	miRNA	Mature sequence	Genomic coordinate^b^	Genomic location^b^	Sugarcane EST
miR156	miR156a^a^	gcucacucucuaucugucagc	3:3473045.0.3473132:−	Intergenic	SCQGLV1015B11.g
	miR156b	gcucacuucucuuucugucagc	4:5373544.0.5373631:−	Intergenic	No
	miR156e	gcucgcuucucuuucugucagc	10:55009890.0.55009977:+	Intergenic	No
miR159	miR159b	cuuggauugaagggagcucc	3:1225075.0.1225121:−	Exon	SCAGFL3025B10.g
miR160	miR160a	ugccuggcucccuguaugcca	4:4236166.0.4236252:−	Intergenic	No
miR164	miR164b	uggagaagcagggcacgugcu	4:64881688.0.64881762:‐	Exon	No
miR166	miR166b	ucggaccaggcuucauucccc	1:7426523.0.7426592:+	Intron	SCQGAD1065C10.g
	miR166d	ucggaccaggcuucauucccc	4:63283311.0.63283396:−	Intergenic	SCQGAD1065C12.g
miR167	miR167b^a^	ugaagcugccagcaugaucuga	3:64088380.0.64088466:−	Intergenic	SCSFSD1065B12.g
miR168	miR168a^a^	ucgcuuggugcagaucgggac	4:2246332.0.2246401:‐	utr	SCEPRZ3087H11.g
miR171	miR171a	ugauugagccgugccaauauc	1:7845729.0.7845804:−	Intergenic	No
	miR171c	ugagccgagccaauaucacuuc	2:17125742.0.17125820:−	Intergenic	No
	miR171e	ugagccgaaccaauaucacuc	6:54609050.0.54609135:+	Intergenic	No
	miR171f	ugagccgaaccaauaucacuc	4:62099920.0.62099999:−	Intergenic	No
	miR171h	uugagccgcgucaauaucucc	1:15608733.0.15608816:−	Intergenic	No
	miR171i	ugauugagccgugccaauauc	1:52558150.0.52558237:−	Intergenic	No
	miR171j	uugagccgcgccaauaucucu	10:54088664.0.54088747:+	Intergenic	SCJFAD1013C10.g
	miR171k	ugauugagccgugccaauauc	6:57730667.0.57730744:−	Intergenic	No
miR172	miR172e	ugaaucuugaugaugcugcac	2:14181333.0.14181407:−	Intergenic	No
miR393	miR393b	ucagugcaaucccuuuggaau	6:61406226.0.61406311:−	Intergenic	No
miR394	miR394a	uuggcauucuguccaccucc	2:66910981.0.66911054:+	Intergenic	SCSBFL1108F06.g
miR395	miR395b	guucucugcaagcacuucacg	6:58761024.0.58761088:+	Intergenic	No
	miR395c	guucccuacaagcacuucacg	6:58197026.0.58197095:−	Intergenic	No
	miR395e	guucucugcaagcacuucacg	6:58197552.0.58197616:−	Intergenic	No
	miR395f	ugaaguguuugggggaacuc	6:58196851.0.58196932:‐	Intergenic	SCSGAD1006A12.g
	miR395h	guucccuucaagcacuucaca	6:58761342.0.58761423:+	Intergenic	No
	miR395j	guucccuucaagcacuucaca	7:4658065.0.4658152:+	Intergenic	No
	miR395l	guucccuucaagcacuucaca	7:4658541.0.4658625:+	Intergenic	No
miR396	miR396^a^	uuccacagcuuucuugaacug	4:66092530.0.66092618:−	Intron	SCCCCL7C05F04.g
miR397	miR397−5p	ucaccggcgcugcacucaauu	4:4027096.0.4027184:−	Exon	No
miR399	miR399b	gugcagcucuccucuggcaug	4:9842733.0.9842814:−	Intergenic	No
	miR399i	ugccaaaggagaguugcccug	6:55042944.0.55043024:+	Intergenic	No
	miR399j	ugccaaaggagaauugcccug	4:9862937.0.9863027:−	Intergenic	No
	miR399k	ugccaaaggggauuugcccgg	4:9868286.0.9868347:+	Intron	No
miR528	miR528^a^	uggaaggggcaugcagaggag	1:71476710.0.71476794:−	Intergenic	SCUTSD1026H02.g
miR2118	miR2118−5p	ggcaugggaacauguaggaagg	6:46386348.0.46386421:−	Intergenic	No
miR6222	miR6222−3p	uagcugauccaaacaggcccu	1:40668641.0.40668705:−	Intergenic	SCEZRT3070B02.g^c^
	miR6222−5p	uagcugauccaaacaggcccu	1:40668684.0.40668772:‐	Intergenic	SCEZRT3070B02.g^c^
miR6223	miR6223−5p	cuagcauguuccuccuaagag	7:8092842.0.8092921:+	Exon	No

a miRNAs previously identified for sugarcane and deposited onto miRBase v.21;

b Genome coordinate and location according to *S. bicolor* v. 3.1;

c Corresponds to the same EST.

By mapping miRNAs onto *S. bicolor* genome, it was observed that the majority of the miRNAs are predicted to be encoded by an independent transcriptional unit (intergenic genomic location), whereas only 8 are located into gene sequences (exon, intron, or utr according to *S. bicolor* genomic location) (Table 1). Half of those are predicted to be encoded by mRNA coding sequences. Thirteen out of 39 miRNAs were assigned to putative correspondent sugarcane ESTs, but 2 miR6222 coincided with the same EST. Seven ESTs correspond to 8 miRNAs not yet deposited into miRBase. miR159b, comprised within EST SCAGFL3025B10.g sequence, was one of the few precursors predicted to be encoded by an exon (according to *S. bicolor* genomic coordinates).

### 3.3 miRNA expression profiles and target prediction by using *A. thaliana* genome and sugarcane EST as queries

**Figure 3:**
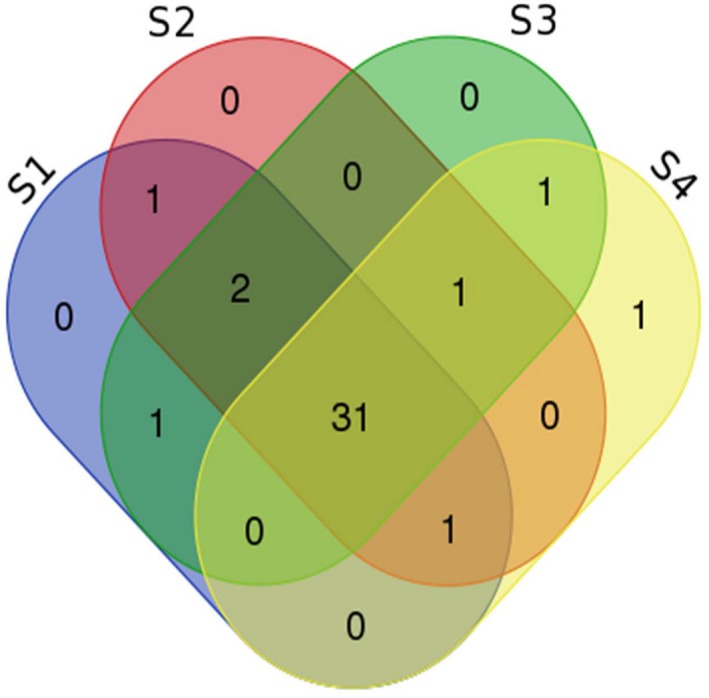
Distribution of the number of shared and exclusive expressed miRNAs during aerenchyma formation in sugarcane root segments (S1 to S4)

**Figure 4:**
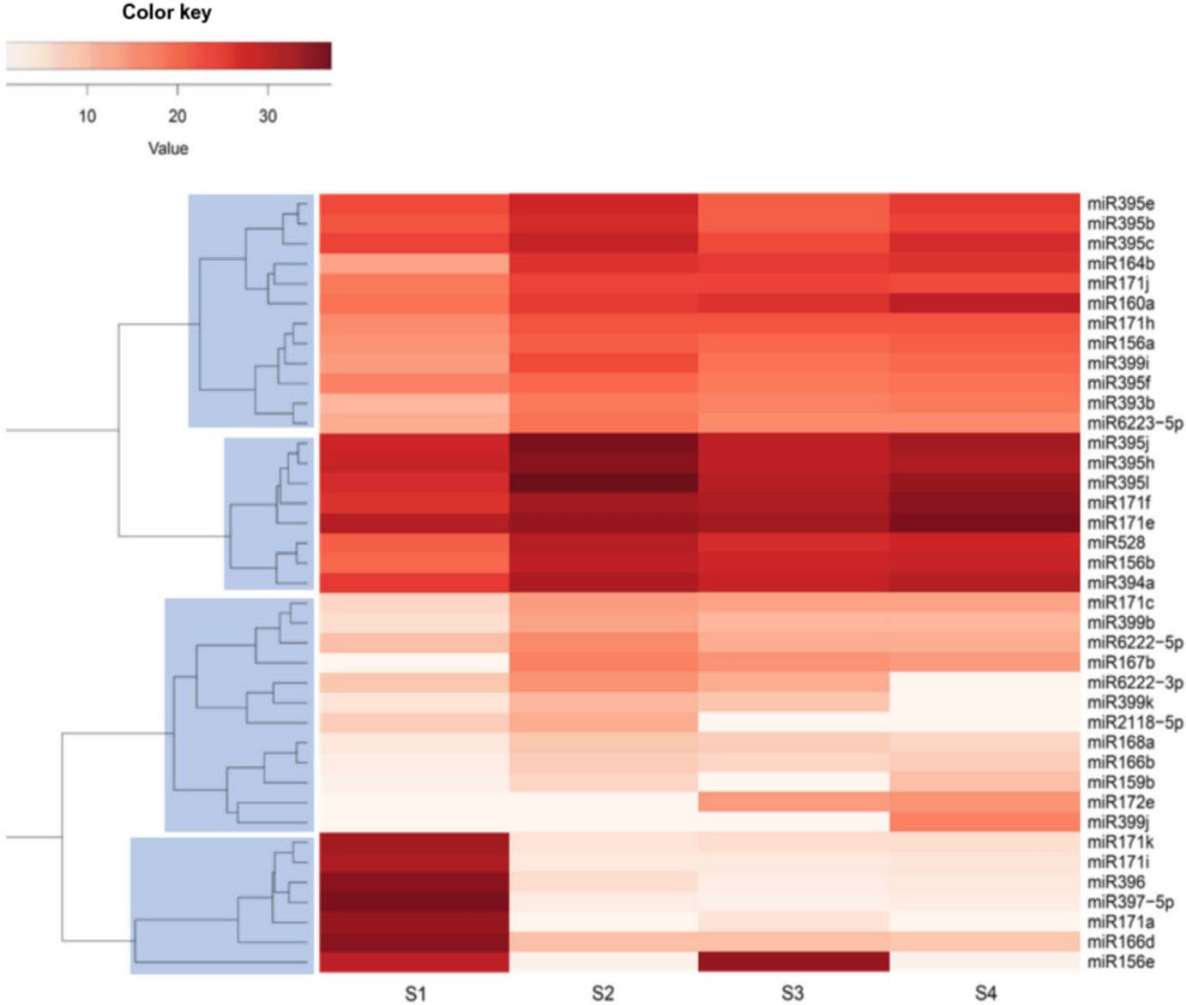
Hierarchical heatmap clustering miRNA expression level along with the sugarcane root segments during aerenchyma formation (S1 to S4). Blue boxes enclose the four major expression clusters

The majority of the identified mature sequences were expressed in all root segments (Figure 3), which represents more than 86% of the total miRNA identified in the present study. This indicates that miRNAs are present during aerenchyma development. The expression level in terms of reads per million (Figure 4 and Table S4) shows four general expression patterns regarding the range of expression (blue boxes). However, there is an overall tendency of lower miRNA expression levels on S1, except for the most basal clade. Furthermore, miRNAs from the same family do not present similar expression levels.

The set of identified miRNA was used to search for predicted targets by using as queries the SUCEST database and the *A. thaliana* genome. Putative targets were predicted for all the 39 miRNAs identified in the present study [747 (Table S5) and 4,728 (Table S6) targets] for SUCEST and *A. thaliana* genome, respectively. In most cases, the predicted targets are inhibited by cleavage instead of translation blockage. Five miRNAs and 8 putative targets were selected for qRT‐PCR validation (Tables S7 and S8), but there were no significant differences among root segments using one‐way ANOVA.

**Figure 5:**
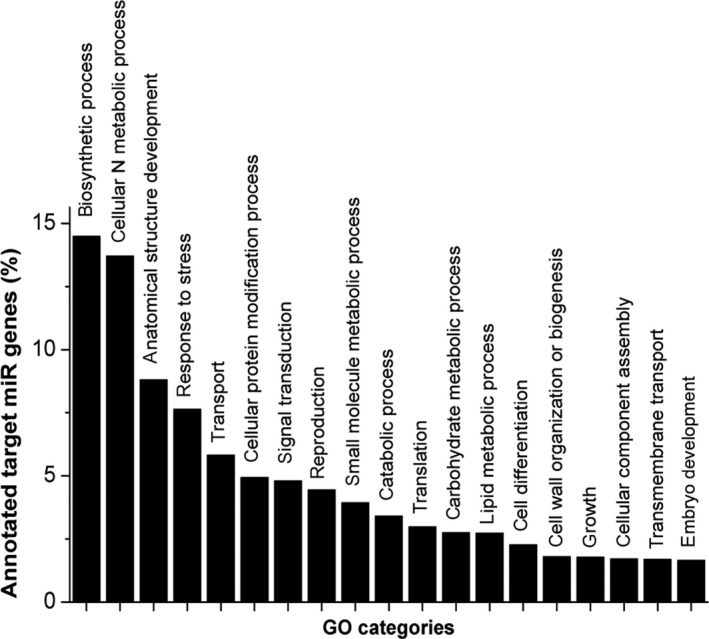
Gene ontology usage of miRNA targets. Only the 19 first categories are shown. Bars indicate the percentage of annotated GO terms for predicted targets of sugarcane miRNA using *Arabidopsis thaliana *as a query

Gene ontology term enrichment for the complete set of putative targets identified in *A. thaliana* recovered several enriched functional categories and processes, including biosynthetic process (14.5%), cellular nitrogen compound metabolic process (13.7%), anatomical structure development (8.8%), response to stress (7.6%), and transport (5.8%) (Figure 5 and Table S9). Cell wall organization or biogenesis and carbohydrate metabolic process were the 15th and 12th most represented categories, with 2.3% and 3.5% of assigned targets respectively.

All of the hydrolysis modules (cell expansion, cell separation, hemicellulose, and cellulose hydrolyzes) directly related to the cell wall (Grandis et al., 2014) are predicted to be targeted by miRNAs (Table 2). Cell separation, which comprises pectin hydrolysis, presented the highest number and diversity of predicted targets (50%), followed by hemicellulose hydrolysis (22.4%), cellulose modifications (15.5%), and cell expansion (12.1%). Remarkably, it was observed that pectins are indeed somehow subjected to structural changes during aerenchyma formation in sugarcane (Leite et al., 2017). This is a committed step for the attack of specific enzymes to the other polysaccharides, hemicelluloses, and cellulose that culminates in aerenchyma formation (Tavares et al., 2015, 2019) and production of a cell wall composite (Grandis et al., 2019; Leite et al., 2017).

**Table 2:** Predicted cell wall‐related transcripts as putative targets for sugarcane miRNAs using *Arabidopsis thaliana* genome and SUCEST database as queries. The targets were distributed among cell wall modules according to Grandis et al. (2014)

Module	Target description	CAZy or annotation	Target transcript (miRNA)
Cell expansion	Expansin	Expansin A17	AT4G01630 (miR6222−3p, miR6222−5p)
	Extensin	(Pro‐rich) extensin‐like; Pro‐rich extensin receptor kinase	AT4G13340 (miR397−5p); AT3G22800 (miR171a, miR171i, miR171k); AT4G08410 (miR395c, miR395h, miR395j, miR395l); AT5G59170 (miR393b); AT3G24550 (miR171c); AT2G18470 (miR6222−3p, miR6222−5p)
Cell separation	Pectate lyase	PL1_1	AT4G22090 (miR394a); AT5G15110 (miR168a); AT5G55720 (miR171a, miR171i); AT3G55140 (miR168a, miR2118−5p)
	Pectin lyase‐like protein	GH28	AT1G43100 (miR2118−5p)
	Pectin methylesterase	CE8	AT3G17060 (miR171j, miR2118−5p); AT2G21610 (miR159b); AT4G00190 (miR167b); AT1G44980 (miR397−5p)
	Pectin methylesterase inhibitor	PMEI2	AT3G17220 (miR399i, miR2118−5p)
	Pectin acetyl esterase	CE13	AT3G05910 (miR6222−3p, miR6222−5p)
	Polygalacturonase	GH28, PG1	AT4G20040 (miR159b); AT1G43100 (miR2118−5p); AT1G17150 (miR528); AT1G60390 (miR395b, miR395e); AT3G07970 (miR395h, miR395j, miR395l); AT4G01890 (miR167b); AT4G23820 (miR395f)
	β‐galactosidase	GH35; β‐gal‐related protein	AT4G38590 (miR395f); AT3G52840 (miR156a); AT4G36360 (miR395b, miR395e); AT5G63800 (miR2118−5p); AT5G49250 (miR166b, miR166d)
	Fasciclin‐like arabinogalactan protein	FLA1; FLA3; FLA8; FLA14; FLA17	AT5G55730 (miR171a, miR171i, miR171k); AT5G44130 (miR166b, miR166d); AT3G12660 (miR156e); AT5G06390 (miR395b, miR395e); AT2G24450 (miR395f); AT2G45470 (miR396)
Hemicellulose hydrolysis	Mannosidase	GH5	AT4G28320 (miR395h, miR395j, miR395l)
	Feruloyl esterase	Putative esterase‐like	AT5G11910 (miR6222−3p, miR6222−5p)
	Fucosidase	α‐L‐fucosidase 2	SCACLR1036F01.g (miR6223−5p)
	β−1,3‐glucosidase	GH17; GH3	AT1G77780 (miR156e); AT5G20950 (miR395f); AT4G18340 (miR171a, miR171i, miR171j, miR171k); AT1G32860 (miR166b, miR166d); AT3G57270 (miR171a, miR171i, miR171k); SCEZLB1006G08.g (miR395b, miR395l); SCCCSD2003H02.g (miR159b); SCEZRT2022D09.g (miR395f)
	Xyloglucan endotransglucosylase/ hydrolase	GH16	AT4G30290 (miR167b)
	Xyloglucanase	GT77	AT2G35610 (miR399i)
Cellulose hydrolysis	Endo‐β‐glucanase	GH5; GH5_11; GH9	AT1G19940 (miR6223−5p); AT2G44540 (miR171a, miR171i, miR171k); AT2G44550 (miR171a, miR171i, miR171k); AT2G44560 (miR171a, miR171i, miR171k); AT3G26140 (miR6223−5p); AT1G22880 (miR395f)
	β −1,4‐glucosidase	GH1	AT3G18080 (miR159b); AT1G26560 (miR171e, miR171f); AT5G54570 (miR156b, miR156e)

Besides cell wall modifications, ethylene regulation is also one of the hallmarks underlying aerenchyma formation (Tavares et al., 2019; Yamauchi et al., 2013). Target prediction analysis showed that virtually all steps from ethylene production to transcriptional activity mediated by this phytohormone could be negatively regulated by a subset of the identified miRNAs (Table 3). Eleven families of ethylene‐responsive factors (ERFs) are predicted targets for 11 families of miRNAs. Although they are different subfamilies, presenting at least one AP2 DNA‐binding domain (Licausi, Ohme‐Takagi, & Perata, 2013), they are not all involved in ethylene signal transduction. Indeed, it has been observed that CYTOKININ RESPONSE FACTOR (CRF) and ABA REPRESSOR (ABR) mediate transcriptional response to cytokinin and abscisic acid, respectively (Pandey et al., 2005; Rashotte et al., 2006). Furthermore, most of the ERFs predicted to be negatively regulated by miRNAs display a repression domain within the protein sequence (Licausi et al., 2013).

**Table 3:** Predicted ethylene‐related transcripts as putative targets for sugarcane miRNAs using *Arabidopsis thaliana* genome and SUCEST database as queries

Category	Target description	Target	Target transcript (miRNA)
Ethylene overproduction protein	ETO	ETO1	AT3G51770 (miR396)
Ethylene perception	ERS	ERS2	AT1G04310 (miR171j)
Ethylene signaling	ETHYLENE INSENSITIVE	EIN3	AT3G20770 (miR172e)
Ethylene‐responsive transcription factor	WAX INDUCER	WIN1	AT1G15360 (miR6223−5p)
	RELATIVE TO AB3/VP1	RAV1	AT1G51120 (miR156b)
	Ethylene response factors	ERF	AT1G68550 (miR394a); AT1G71130 (miR528); AT2G20880 (miR395f); AT1G22810 (miR395f); AT2G31230 (miR528); AT4G17490 (miR528); AT4G18450 (miR166b, miR166d); AT5G07580 (miR395f); AT5G21960 (miR166b, miR166d); AT5G47230 (miR528); AT2G41710 (miR171h); AT1G16060 (miR394a); SCUTLR1058E01.g (miR172e); SCVPCL6044F03.g (miR172e);
	Floral homeotic protein APETALA 2	AP2	AT4G36920 (miR172e)
	SCHNARCHZAPFEN	SNZ	AT2G39250 (miR172e)
	WRINKLED	WRI1; WRI4	AT3G54320 (miR164b); AT1G79700 (miR164b)
	SCHLAFMUTZE	SMZ	AT3G54990 (miR172e)
	CYTOKININ RESPONSE FACTOR	CRF6; CRF2	AT3G61630 (miR171h); AT4G23750 (miR164b)
	AINTEGUMENTA‐LIKE	AIL5	AT5G57390 (miR156b)
	TARGET OF EAT	TOE2; TOE3	AT5G60120 (miR172e); SCSBHR1050A07.g (miR172e)
	ABA REPRESSOR	ABR1	AT5G64750 (miR393b)

**Figure 6:**
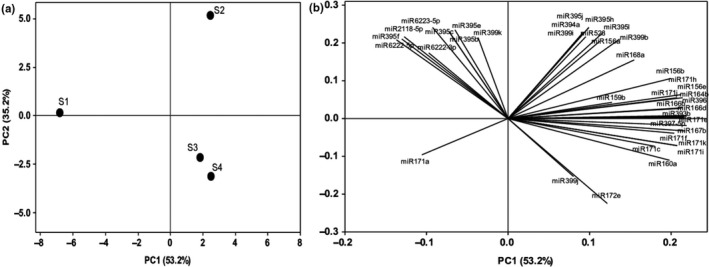
Distance biplots from 39 miRNAs found in sugarcane roots segments during aerenchyma formation. (a) The centroid separation corresponds to data average for roots segments in the plane defined by the first and second main components (PC1 and PC2). Percentage values in parentheses (*x* and *y* axes) show the proportion of the variance explained by axis. (b) The plot of the PC1 and PC2 loading vectors, describing the relationship among the miRNAs and roots segments. The mRNAs analyzed from Table 1 were expressed in descriptor vectors all PCs loading from PC1 and PC2 showed in Table S10. (*n* = 3)

In order to have an overview of miRNA profiles along with the different root segments, a principal component analysis (PCA) was performed (Figure 6a). The PCA allowed for quite clear discrimination among samples, with PC1 (accounting for 53.2% of variation) separating S1 from the other root segments and PC2 (35.2% of variation) splitting root segments consistently with the order of aerenchyma formation, that is, from S1 to S4. The miRNA species that most contributed to the separation of S1 samples in PC1 were miR6222‐5p, miR395f, and miR2118‐5p, whereas miR396, miR166b and miR166d, miR393b, miR397‐5p, miR167b, miR164b, and some miR171 (e, h, and j) were important for segregating samples from the other segments (Figure 6b and Table S10). In PC2, miR172e and miR399j influenced the separation of S1 and S2, while miR528, miR6223‐5p, and most of the members of the miR395 family were important for segregating S3 and S4 (Figure 6b and Table S10). Although it is difficult to find clear patterns of miRNA expression among the identified miRNA members/families and segments (Figure 4), the PCA confirms that their global expression differs along the aerenchyma formation. Indeed, S1 is the meristem, S2 is the beginning of the transformation of the parenchymatic cells into aerenchyma, and S3 and S4 are tissues already displaying aerenchyma regions and therefore the action of the cell wall degrading enzymes.

## 4 DISCUSSION

Several publications reported the identification of miRNAs in biofuel crops such as *Miscanthus giganteus* (Jha & Shankar, 2013), *Setaria italica* (Yadav, Muthamilarasan, Pandey, Khan, & Prasad, 2014; Yi, Xie, Liu, Qi, & Yu, 2013), *Sorghum bicolor* (Calviño, Bruggmann, & Messing, 2011), *Z. mays* (Aravind et al., 2017; Liu et al., 2019), and *Saccharum* sp. (Gentile, Dias, Mattos, Ferreira, & Menossi, 2015 and references therein; Li et al., 2017; Thiebaut et al., 2017; Su et al., 2017; Yang et al., 2017). However, only a few of them addressed specific miRNAs predicted to be involved in cell wall construction or remodeling. In the present work, we identified 39 miRNAs expressed within the roots of the biofuel crop sugarcane undergoing cell wall changes due to aerenchyma formation. In this fine‐tuned process, several classes of enzymes act in a coordinated fashion on the wall polysaccharides of cortex cells to modify and/or degrade them, so that the gas spaces in the roots are formed (Grandis et al., 2019; Leite et al., 2017; Rahji et al., 2011). The identified miRNAs are comprised within 19 families, which are comparable to sugarcane miRNA previously published data [14 (Zanca et al., 2010), 18 (Ferreira et al., 2012), 25 (Carnavale‐Bottino et al., 2013), and 26 (Ortiz‐Morea et al., 2013)]. From the 39 miRNAs, 31 have not yet been identified for sugarcane according to miRBase database release v. 21, but were previously reported in the close relative species *S. bicolor* (Table 1).

The majority of mature sequences presented 21 nt length and U as the 5’ terminal nucleotide (Table S3). The identity of the first nucleotide within a mature sequence represents an important issue regarding DCL cleavage. Usually, 21‐nt‐long mature sequences are a product of DCL1 or DCL4 (Voinnet, 2009). One of the nuclease domains, RNase III‐A, releases preferentially miRNAs with U as the 5’ terminal nucleotide (Starega‐Roslan, Galka‐Marciniak, & Krzyzosiak, 2015). Furthermore, miRNAs loading onto the Argonaute are also directed by the identity of the 5’ nucleotide in plants (Mi et al., 2008), humans (Frank, Sonenberg, & Nagar, 2010), and flies (Okamura, Liu, & Lai, 2009). The strong dominance of 21 nt 5’ U sequences has also been observed for other unrelated species such as *A. thaliana* (Rajagopalan, Vaucheret, Trejo, & Bartel, 2006), *Physcomytrella patens* (Cho et al., 2008), *Z. mays* (Nobuta et al., 2008), and *Populus trichocarpa* (Klevebring et al., 2009)*.*

The knowledge about ethylene signaling makes aerenchyma formation an interesting subject for miRNA transcriptomic studies. Although ethylene has been pointed out as the major hormonal trigger of aerenchyma formation, its levels decrease as the aerenchyma develops (Tavares et al., 2018). Key players of the signal cascade have been described (He, Morgan, & Drew, 1996) and one scERF1 named scRAV1 acting as negative regulator upon pectin hydrolysis at the onset of aerenchyma formation was identified (Tavares et al., 2019). miR156 and miR172 are known negative regulators of AP2/ERF transcription factors implicated in flower organogenesis (Yant et al., 2010), developmental timing (Wu et al., 2009), nodulation (Nova‐Franco et al., 2015; Wang et al., 2014), ripening (Bi, Meng, Ma, & Yi, 2015), and apical dominance (Schwab et al., 2005). Thirteen miRNAs were predicted to target transcripts related to ethylene production, perception, signal transduction, and ethylene‐mediated transcriptional activity (Table 3). Among those, miR172, which was only detected when the aerenchyma is already visible (S3 and S4), targets transcripts from the last two of the mentioned categories. As a consequence, the role played by the targeted ERFs might take place at the onset of aerenchyma formation (S1 and S2), what is expected according to previous observations of our group using the same type of experiment (Tavares et al., 2019). At this point, miR172 transcription is rather low or absent, and cell wall changes are not yet visible (Figure 4). *EIN3* is a known positive component in ethylene response (Takahashi, Yamauchi, Rajhi, Nishizawa, & Nakazono, 2015; Tavares et al., 2019). It is possible that EIN3 is not repressed by miR172 at the initial stages of aerenchyma development, and when PCD starts, the expression levels of miRNA172 increase and repress EIN3 in S3 and S4. This would possibly happen after ethylene signaling to PCD.

It has been hypothesized that cell wall modifications rely on a general succession of events named modules (Grandis et al., 2014). Here, we observed that cell wall organization or biogenesis and carbohydrate metabolic process were well‐represented ontology categories among putative miRNA target genes (Figure 5 and Table S9). Those include transcripts related to cell expansion and separation, and cellulose and hemicellulose hydrolysis modules. Thirty‐one out of 39 miRNAs are predicted to target at least one cell wall‐modification‐related transcript (Table 2). miRNA6222‐3p (Tables S5 and S6) stands out for targeting transcripts coding for 5 different enzyme activities. Except for cellulose hydrolysis, all other cell wall degradation modules are predicted to be targeted by this miRNA. In addition, all degradation modules are targeted by members from miR171 and miR395 families (Table 2).

Hemicellulose hydrolysis‐related targets are around 22% of cell wall‐related transcripts targeted by miRNA during aerenchyma formation. Arabinoxylan debranching and β‐glucan hydrolysis occur during this process (Grandis et al., 2019; Leite et al., 2017). miR6223‐5p showed decreasing levels along the root segments, which suggests that the targeted feruloyl esterase mRNA would be de‐repressed as the aerenchyma develops. This esterase breaks ferulic acid bridges between arabinose residues that decorate arabinoxylan backbones. Besides being responsible for the cross‐linking among arabinoxylan chains, ferulic bridges may also anchor lignin to arabinoxylan in grasses (Buanafina, 2009). As a result, when ferulic acid is absent, arabinosyl residues can be further hydrolyzed, allowing the enzyme attack on xylan. Consistent with this, the levels of arabinose decrease from S1 to S4, whereas the lignin content remains stable along the root (Leite et al., 2017). Thus, the control of expression of feruloyl esterases by miRNAs might be important because it is likely to promote loosening of interchain connections. Such modification would allow the action of hydrolases, producing the composite that confers impermeability to gasses in the aerenchyma (Leite et al., 2017).

Cell separation encompasses half of the targeted cell wall‐related transcripts (Table 2) and mostly relies on pectin degradation (Grandis et al., 2019; Leite et al., 2017; Tavares et al., 2019). This module includes carbohydrate‐active enzymes such as glycosyl hydrolases, esterases, and lyases (Grandis et al., 2014, 2019).

The most abundant pectin in plant cell walls is homogalacturonan and its de‐acetylation and de‐methylation by pectin acetyl and methylesterases, respectively. This occurs prior to the attack on α‐1,4 linkages between galacturonic acids by endopolygalacturonases or by eliminative cleavage by pectate lyase (Kohli & Gupta, 2015; Latarullo et al., 2016; Yadav, Yadav, Yadav, & Yadav, 2009). β‐galactosidase is also thought to increase cell wall porosity through the hydrolysis of galactan side chains from heavily decorated pectin (Grandis et al., 2019; Leite et al., 2017). Transcripts coding for all 5 enzyme activities are among putative targets for miRNA expressed during aerenchyma formation (Table 2).

Some of the pectin degradation‐related targets of miRNA regulation have been reported elsewhere, in accordance with what we observed in sugarcane roots. miR156, miR167, miR171, miR394, miR395, and miR2118 are expressed in *Panicum virgatum*, lotus, and cotton when subjected to abiotic stress. These miRNAs were predicted to target pectinesterase, β‐galactosidase, pectin lyase, and endopolygalacturonase mRNAs (Xie et al., 2014; Xie, Wang, Sun, & Zhang, 2015). Also, overexpression of miR156 in alfalfa led to disturbed pectin content (Aung et al., 2015). These findings, together with the observation by Leite et al. (2017) that galactose consistently decreases and β‐galactosidase increases (Grandis et al., 2019) during aerenchyma formation are consistent with the hypothesis that miR156 might be a negative regulator of pectin degradation through β‐galactosidase within sugarcane roots ongoing aerenchyma formation.

The cell separation module comprises not only pectinases but also fasciclin‐like arabinogalactan proteins. They are a class of hydroxyproline‐rich proteins highly abundant within cell walls and plasma membrane (Harpaz‐Saad et al., 2011; Shi, Kim, Guo, Stevenson, & Zhu, 2003). Ethylene precursor 1‐aminocyclopropane‐1‐carboxylic acid is a putative key mediator of the role played by fasciclin‐like proteins upon cell wall regulation within roots (Xu, Rahman, Baskin, & Kieber, 2008). These proteins have also been implicated in programmed cell death (Gao & Showalter, 1999), which is a feature of aerenchyma formation in sugarcane roots (Leite et al., 2017). Fasciclin‐like arabinogalactan‐related mRNAs alone were predicted to be targeted by 12 miRNAs. It has been suggested that the role played by β‐galactosidases on pectin porosity could be mediated by the interaction with arabinogalactan‐like proteins due to the removal of galactosyl side chains (Dean et al., 2007; Lamport, Kieliszewski, & Showalter, 2006; Seifert & Roberts, 2007). The transcriptional pattern of miR171i, miR171k, and miR166d displayed a tendency to increase along the sugarcane root segments (Figure 4). This might suggest enhanced translational blockage of the putative targets β‐galactosidase and fasciclin‐like arabinogalactan mRNAs. The lower miRNA levels closer to the root apex are consistent with the expected role played by β‐galactosidase and fasciclin arabinogalactan‐like proteins prior to major cell wall changes. Altogether, miRNA transcription activity supports the idea of pectin porosity (Baron‐Epel, Gharyal, & Schindler, 1988) as a preparatory step for further cell wall modifications during aerenchyma formation in sugarcane roots. Hypothetically, an increase in porosity due to the action of β‐galactosidase on the walls of sugarcane roots, especially at S2, might be responsible for opening the access to other wall‐modifying enzymes and produce the composite that is the final product of the aerenchyma formation process (Grandis et al., 2019; Leite et al., 2017).

Endopolygalacturonase is also a key enzyme for aerenchyma development in sugarcane roots (Tavares et al., 2019). Endopolygalacturonase mRNA levels increase upon transcriptional de‐repression by scRAV1 at the same time as ethylene declines, and major changes regarding pectins become visible (Leite et al., 2017; Tavares et al., 2018). Curiously, the blockage of ethylene perception using the drug 1‐methylcyclopropene (1‐MCP) delays but is unable to prevent aerenchyma development (Tavares et al., 2018). miR6223‐5p, miR528, and miR2118 decreasing levels from S1 to S4 might indicate that translational activity upon target transcripts (pectin acetyl esterase and endopolygalacturonase) is de‐repressed (Figure 4). These results suggest that, besides the hormone and transcriptional repression played by ethylene through scRAV1, endopolygalacturonase hydrolysis could also be subjected to miRNA regulation. Furthermore, the connection between ethylene perception and action, and pectin degradation through scRAV repression might also be influenced by miRNA regulation. The present work showed that miR156 is predicted to target scRAV1 (Tavares et al., 2019) transcription factor. This result is consistent with the observation that RAV1 expression was affected under miR156 overexpression in rice (Xie et al., 2012). Moreover, alteration in this miRNA led to a reduction in lignin content, higher cellulose accumulation, increased sugar yield, and improved digestibility in *P. virgatum*, maize, alfalfa, and poplar (Chuck et al., 2011; Fu et al., 2012; Rubinelli, Chuck, Li, & Meilan, 2013). miR156‐overexpressing plants of *Medicago sativa* were shown to have contrasting expression patterns for different endopolygalacturonases (Gao, Austin, Amyot, & Hannoufa, 2016). Whether miR156 regulates pectinase genes directly or via RAV1 inhibition remains to be solved.

The global analysis of miRNA expression levels revealed a clear distinction among all root segments, and S1 was completely separated from S2, S3, and S4 in PC1 (Figure 6). Several miRNAs influenced this separation (Table S10), but, probably, they are more related to the different developmental root zones rather than with the formation of aerenchyma itself. PC2 discriminates S2 completely from the other segments, suggesting that this segment is where most of the epigenetic control via miRNA of aerenchyma formation takes place in sugarcane roots. S3 and S4 were closely grouped, with miR172e and miR399j being relevant in this separation. In these segments, the ethylene signaling, programmed cell death, and pectin degradation modules are already advanced, and the formation of air spaces is visible (Leite et al., 2017). The miRNA expression levels alone (Figure 4) make it difficult to define clear patterns able to explain the aerenchyma formation. However, the global analysis reinforces the fact that the segments differ among each other and miRNA expression possibly contributes to this process.

## 5 CONCLUSIONS

Our results show that miRNA epigenetic control in sugarcane is predicted to target several genes within a range of GO categories, including biosynthetic process, cellular N metabolic process, anatomical structure development, to name but a few. Our analyses, focused on the cell wall‐ and hormone‐related processes in the roots of sugarcane, suggest the existence of a layer of epigenetic control via miRNA. Considering the modularity of aerenchyma formation (ethylene action, cell expansion, cell separation, hemicellulose, and cellulose hydrolysis), the sugarcane miRNAs found in this work seem to be involved in hormone perception, cell separation, and hemicellulose hydrolysis.

Since sugarcane is an important bioenergy crop, our findings may have some impact on the production of bioenergy in the future, as the aerenchyma formation process offers opportunities to gain control of the whole‐plant cell wall hydrolysis and biomass saccharification.

## CONFLICT OF INTEREST

The authors declare no conflict of interest associated with the work described in this manuscript.

## AUTHOR CONTRIBUTIONS

E.Q.P.T performed most of the experiments; E.Q.P.T, G.H.R, and M.S.B designed the experiments and analyzed the data; A.R.P and J.W.G provided technical assistance with bioinformatic analysis and figure design; E.Q.P.T and M.S.B conceived the project and wrote the manuscript; A.G and M.C.M.M incorporated new data analyses; A.G, M.C.M.M, and M.S.B rewrote the manuscript; and M.S.B agrees to serve as the author responsible for contact and ensures communication.
