# Supplementary material for: Newly identified miRNAs may contribute to aerenchyma formation in sugarcane roots
Source: Plant Direct. 2020 Mar 26;4(3):e00204. doi: 10.1002/pld3.204 (PMC7098396; doi:10.1002/pld3.204)
Supplement: Supplementary file 1 [file PLD3-4-e00204-s001.docx]

**Supplemental Table S1.**

| **Oligonucleotide (Target)** | **SAS - Sucest** | **Forward primer** | | **Reverse primer** | | **Stem loop primer** | |  |
| --- | --- | --- | --- | --- | --- | --- | --- | --- |
| Universal_R | - |  | | CGGTCGTCGTATCCAGTGCG | |  | |  |
| miR156 a | - | GCTCACTCTCTATCTGTCAGCGT | |  | | GTCGTATCCAGTGCAGGGTCCGAGGTATTCGCACTGGATACGACGCTGAC | |  |
| miR6223 -5p | - | CTAGCATGTTCCTCCTAAGAGGTCG | |  | | GTCGTATCCAGTGCAGGGTCCGAGGTATTCGCACTGGATACGACCTCTTA | |  |
| miR395 a,b,f,j,l | - | TGAAGTGTTTGGGGGAACTCGT | |  | | GTCGTATCCAGTGCAGGGTCCGAGGTATTCGCACTGGATACGACGAGTTC | |  |
| miR164 b | - | TGGAGAAGCAGGGCACGT | |  | | GTCGTATCCAGTGCAGGGTCCGAGGTATTCGCACTGGATACGACAGCACG | |  |
| miR399 a,b,I,k | - | TGCCAAAGGAGAGTTGCCCTG | |  | | GTCGTATCCAGTGCAGGGTCCGAGGTATTCGCACTGGATACGACCAGGGC | |  |
| HOX 1 (miRNA166) | SCCCLR1022C07 | TCCAAGGCGACTGGAACTGC | | TGGGCTCAAGGCTCACAAGG | |  | |  |
| NAC (miRNA164b) | SCEPRT2048G05 | TGGCATGAGCTACCTGGACCAT | | TTCTTGTCGCTGTTGAGAGGATCA | |  | |  |
| APETALA 2 (miRNA172e) | SCJLRT1022F08 | AGCACGAAGGCAAAGCTGGA | | TTGCAAGGCAGGTGGAGGAG | |  | |  |
| HOX 2 (miRNA166) | SCQSAM2100G05 | TGCAAATGGTTGGGATGAAGC | | GCGACCTTTGTGGGCTCAAG | |  | |  |
| HOX 3 (miRNA166) | SCRFLR1034E12 | GAGGTGGCTCCATGATTCACA | | CCTCAGGCACACTCCAAGCA | |  | |  |
| GT 8 (miRNA164) | SCRLLR1016F07 | ATCCCCAGGTGGATTCAGCA | | CGGACAATGTTTTGGCAGCA | |  | |  |
| ERF RAP2-7 (miRNA172e) | SCSBHR1050A07 | AGCTGGGCATGGAAGGTGAC | | TGTCGTGGCGGTATTGGAGA | |  | |  |
| AP2/EREBP (miRNA172e) | SCVPCL6044F03 | GCAACCCCAGCATCAACATC | | CTCTGCCCGTTTCTCCTCCA | |  | |  |
| 60S | SCJFRZ2009G01.g | | GCGAGTGCCTCACCTTTGAC | | TCTTAGGTCCCCTCAGCAGAAC | |  | |
| ACT11 | SCRFLR1012H05.g | | CTGACGCCGAGGATATCC | | CAGCGTAACCCAGCCTTGA | |  | |
| GAPDH | CA254672 | | CACGGCCACTGGAAGCA | | TCCTCAGGGTTCCTGATGCC | |  | |
| PUB | SCCCST2001G02.g | | CCGGTCCTTTAAACCAACTCAGT | | CCCTCTGGTGTACCATTTG | |  | |
| UB | SCBGLR1002D06.g | | GGTGGCCGGCTTGGA | | TTTGTTTCGGTTTCAAGTCGATAA | |  | |
| UBE-2 | SCBGLR1002D06.g | | CAGGTCCTGCTGGTGAGGAT | | CACCTCCAGCATATGGACTATCAG | |  | |
| UBQ-10 | SCCCCL3080A11.g | | CGTCCGCAGTCCCCAAT | | TGAGAGGATCGCGAGGATTC | |  | |

**Supplemental Table S2.**

| **Description** | **S1** | **S2** | **S3** | **S4** | **Total** |
| --- | --- | --- | --- | --- | --- |
| **All reads** | 73,067,435 | 39,230,229 | 36,629,202 | 31,508,346 | 180,435,212 |
| **Filtered reads^*^** | 48,884,411 | 25,896,545 | 24,637,913 | 20,542,748 | 119,961,617 |
| **Identified miRNAs** | 36 | 36 | 36 | 35 | 39 |

**Supplemental Table S3.**

|  | **A** | **C** | **G** | **U** | **ALL** |
| --- | --- | --- | --- | --- | --- |
| 20 | 0.0 | 2.4 | 0.0 | 7.1 | 9.5 |
| 21 | 0.0 | 7.1 | 47.6 | 90.5 | 76.2 |
| 22 | 0.0 | 0.0 | 9.5 | 14.3 | 14.3 |

**Supplemental Table S4.**

| **mature miRBase miRNA** | **S1** | **S2** | **S3** | **S4** |
| --- | --- | --- | --- | --- |
| miR156a | 6,242712 | 6,80579 | 6,544288 | 6,234232 |
| miR156b | 6,758027 | 8,121765 | 7,70224 | 7,561599 |
| miR156e | 8,836217 | 10,28856 | 9,708591 | 10,14613 |
| miR159b | 0,11118 | 1,334861 | 0 | 1,698292 |
| miR160a | 6,697255 | 7,322348 | 7,56415 | 7,820702 |
| miR164b | 6,09116 | 7,462673 | 7,488903 | 7,137239 |
| miR166b | 13,46161 | 14,56638 | 14,24631 | 14,54716 |
| miR166d | 9,621193 | 14,94144 | 14,57318 | 14,77451 |
| miR167b | 0 | 3,515416 | 3,861177 | 3,93276 |
| miR168a | 13,50529 | 14,61007 | 14,29331 | 13,76946 |
| miR171a | 9,072918 | 0 | 11,77728 | 0 |
| miR171c | 2,466346 | 2,9943 | 2,868087 | 3,433715 |
| miR171e | 8,899631 | 9,528556 | 9,400419 | 9,568478 |
| miR171f | 8,582966 | 9,16085 | 9,375446 | 9,162009 |
| miR171h | 6,393144 | 6,830425 | 6,739939 | 6,712779 |
| miR171i | 9,069334 | 10,96374 | 11,77567 | 11,46197 |
| miR171j | 6,522258 | 7,060274 | 6,947057 | 6,939085 |
| miR171k | 9,072828 | 10,96611 | 11,77752 | 11,46408 |
| miR172e | 0 | 0 | 3,583945 | 4,081382 |
| miR2118-5p | 2,790768 | 2,663128 | 0 | 0 |
| miR393b | 4,694296 | 5,543967 | 5,525111 | 5,500633 |
| miR394a | 8,210274 | 8,619862 | 8,162133 | 8,333172 |
| miR395b | 7,58638 | 7,857306 | 6,646796 | 7,030518 |
| miR395c | 7,651261 | 7,951787 | 6,797466 | 7,143566 |
| miR395e | 7,5876 | 7,858013 | 6,646796 | 7,0335 |
| miR395f | 6,4634 | 6,385482 | 5,576269 | 5,665945 |
| miR395h | 8,789951 | 9,547854 | 8,881238 | 8,84922 |
| miR395j | 8,78917 | 9,548205 | 8,881238 | 8,851351 |
| miR395l | 8,737589 | 9,599508 | 8,882026 | 8,902543 |
| miR396 | 9,621193 | 11,20145 | 11,06213 | 11,15378 |
| miR397-5p | 9,804775 | 10,74368 | 10,7536 | 10,80846 |
| miR399b | 2,146504 | 2,97521 | 2,366417 | 2,377326 |
| miR399i | 6,145149 | 6,985352 | 6,471179 | 6,045042 |
| miR399j | 0 | 0 | 0 | 5,14177 |
| miR399k | 1,43467 | 2,071535 | 1,439061 | 0 |
| miR528 | 7,36025 | 8,326534 | 7,697484 | 7,271816 |
| miR6222-3p | 3,173635 | 3,109991 | 2,621181 | 0 |
| miR6222-5p | 3,201352 | 3,12671 | 2,621181 | 2,607838 |
| miR6223-5p | 5,805015 | 6,081635 | 4,636751 | 4,681834 |

**Supplemental Table S5.** (uploaded as sheet file)

**Supplemental Table S6.** (uploaded as sheet file)

**Supplemental Table S7.**

|  | **miR156a** | **miR164b** | **miR395** | **miR399** | **miR6223** |
| --- | --- | --- | --- | --- | --- |
| **S1** | 0.099 ± 0.07 | 0.961 ± 0.47 | 4.635 ± 2.57 | 0.040 ± 0.03 | 0.758 ± 0.42 |
| **S2** | 4.843 ± 2.15 | 2.116 ± 1.24 | 4.789 ± 3.82 | 9.668 ± 6.11 | 1.549 ± 0.51 |
| **S3** | 2.778 ± 0.50 | 1.260 ± 0.55 | 1.161 ± 1.02 | 2.219 ± 1.67 | 1.297 ± 0.55 |
| **S4** | 3.107 ± 0.47 | 1.141 ± 0.49 | 1.180 ± 0.64 | 12.79 ± 3.70 | 2.487 ± 1.26 |
| **R^2^** | \| 0.1988 \| \| --- \| | 0.3746 | 0.2644 | 0.1373 | 0.2440 |

**Supplemental Table S8.**

|  | **APETALA 2** | **AP2/EREBP** | **ERF RAP 2-7** | **GT 8** | **HOX 1** | **HOX 2** | **HOX 3** | **NAC** |
| --- | --- | --- | --- | --- | --- | --- | --- | --- |
| **S1** | 0.69 ± 0.09 | 1.36 ± 0.29 | 0.94 ± 0.09 | 0.83 ± 0.08 | 0.65 ± 0.06 | 0.64 ± 0.09 | 0.60 ± 0.12 | 0.33 ± 0.03 |
| **S2** | 1.08 ± 0.22 | 0.67 ± 0.02 | 1.43 ± 0.24 | 1.13 ± 0.25 | 1.06 ± 0.11 | 1.21 ± 0.18 | 0.93 ± 0.31 | 2.15 ± 0.40 |
| **S3** | 1.19 ± 0.17 | 1.07 ± 0.21 | 0.94 ± 0.11 | 1.24 ± 0.22 | 1.15 ± 0.29 | 1.56 ± 0.45 | 1.30 ± 0.41 | 1.53 ± 0.25 |
| **S4** | 1.25 ± 0.15 | 1.24 ± 0.33 | 0.85 ± 0.11 | 0.96 ± 0.15 | 1.42 ± 0.22 | 1.02 ± 0.23 | 1.95 ± 0.38 | 1.02 ± 0.19 |

**Supplemental Table S9.** (uploaded as sheet file)

**Supplemental Table S10.**

|  | **PC1** | **PC2** |
| --- | --- | --- |
| **Eigenvalue** | 20.744 | 13.747 |
| **Proportion** | 0.532 | 0.352 |
|  |  |  |
|  | **PC1** | **PC2** |
| **miR156a** | 0.112 | 0.204 |
| **miR156b** | 0.2 | 0.106 |
| **miR156e** | 0.208 | 0.055 |
| **miR159b** | 0.128 | 0.044 |
| **miR160a** | 0.199 | -0.11 |
| **miR164b** | 0.21 | 0.028 |
| **miR166b** | 0.214 | 0.028 |
| **miR166d** | 0.219 | 0.006 |
| **miR167b** | 0.218 | -0.032 |
| **miR168a** | 0.155 | 0.155 |
| **miR171a** | -0.106 | -0.096 |
| **miR171c** | 0.18 | -0.073 |
| **miR171e** | 0.216 | 0.005 |
| **miR171f** | 0.204 | -0.039 |
| **miR171h** | 0.212 | 0.064 |
| **miR171i** | 0.207 | -0.072 |
| **miR171j** | 0.215 | 0.055 |
| **miR171k** | 0.207 | -0.071 |
| **miR172e** | 0.121 | -0.225 |
| **miR393b** | 0.219 | 0.002 |
| **miR394a** | 0.096 | 0.217 |
| **miR395b** | -0.072 | 0.231 |
| **miR395c** | -0.065 | 0.235 |
| **miR395e** | -0.072 | 0.231 |
| **miR395f** | -0.13 | 0.21 |
| **miR395h** | 0.099 | 0.241 |
| **miR395j** | 0.099 | 0.241 |
| **miR395l** | 0.116 | 0.228 |
| **miR396** | 0.219 | 0.008 |
| **miR399b** | 0.137 | 0.21 |
| **miR399i** | 0.092 | 0.227 |
| **miR399j** | 0.081 | -0.152 |
| **miR399k** | -0.036 | 0.215 |
| **miR528** | 0.094 | 0.23 |
| **miR397-5p** | 0.219 | -0.019 |
| **miR2118-5p** | -0.127 | 0.218 |
| **miR6222-3p** | -0.097 | 0.175 |
| **miR6222-5p** | -0.137 | 0.209 |
| **miR6223-5p** | -0.092 | 0.242 |
